# Parasternal After Cardiac Surgery (PACS): a prospective, randomised, double-blinded, placebo-controlled trial study protocol for evaluating a continuous bilateral parasternal block with lidocaine after open cardiac surgery through sternotomy

**DOI:** 10.1186/s13063-022-06469-5

**Published:** 2022-06-20

**Authors:** Mark Larsson, Ulrik Sartipy, Anders Franco-Cereceda, Anders Öwall, Jan Jakobsson

**Affiliations:** 1 Department of Molecular Medicine and Surgery, Karolinska Institutet, Stockholm, Sweden; 2 Function Perioperative Medicine and Intensive Care, Section for Cardiothoracic Anaesthesia and Intensive Care, Karolinska University Hospital, Stockholm, Sweden; 3 Department of Cardiothoracic Surgery, Karolinska University Hospital, Stockholm, Sweden; 4 Institution for Clinical Sciences, Karolinska Institutet at Danderyds Hospital, Stockholm, Sweden; 5 Department of Anaesthesia and Intensive Care, Danderyds Hospital, Stockholm, Sweden

**Keywords:** Open cardiac surgery, Sternotomy, Postoperative pain, Parasternal block, Recovery, Lidocaine

## Abstract

**Background:**

Multimodal analgesia that provides optimal pain treatment with minimal side effects is important for optimal recovery after open cardiac surgery. Regional anaesthesia can be used to block noxious nerve signals. Because sternotomy causes considerable pain that lasts several days, a continuous nerve block is advantageous. Previous studies on continuous sternal wound infusion or parasternal blocks with long-acting local anaesthetics have shown mixed results. This study aims to determine whether a continuous bilateral parasternal block with lidocaine, which is a short-acting local anaesthetic that has a favourable safety/toxicity profile, results in effective analgesia. We hypothesise that a 72-hour continuous parasternal block with 0.5% lidocaine at a rate of 7 ml/hour on each side provides effective analgesia and reduces opioid requirement. We will evaluate whether recovery is enhanced.

**Methods:**

In a prospective, randomised, double-blinded manner, 45 patients will receive a continuous parasternal block with either 0.5% lidocaine or saline. The primary endpoint is cumulated intravenous morphine by patient-controlled analgesia at 72 hours. Secondary end-points include the following: (1) the cumulated numerical rating scale (NRS) score recorded three times daily at 72 hours; (2) the cumulated NRS score after two deep breaths three times daily at 72 hours; (3) the NRS score at rest and after two deep breaths at 2, 4, 8 and 12 weeks after surgery; (4) oxycodone requirement at 2, 4, 8 and 12 weeks after surgery; (5) Quality of Recovery-15 score preoperatively compared with that at 24, 48 and 72 hours, and at 2, 4, 8 and 12 weeks after surgery; (6) preoperative peak expiratory flow compared with postoperative daily values for 3 days; and (7) serum concentrations of interleukin-6 and lidocaine at 1, 24, 48 and 72 hours postoperatively compared with preoperative values.

**Discussion:**

Adequate analgesia is important for quality of care and vital to a rapid recovery after cardiac surgery. This study aims to determine whether a continuous parasternal block with a short-acting local anaesthetic improves analgesia and recovery after open cardiac procedures.

**Trial registration:**

The study was registered in the European Clinical Trials Database on 27/9/2019 (registration number: 2018-004672-35).

**Supplementary Information:**

The online version contains supplementary material available at 10.1186/s13063-022-06469-5.

## Administrative information

Note: the numbers in curly brackets in this protocol refer to SPIRIT checklist item numbers. The order of the items has been modified to group similar items (see http://www.equator-network.org/reporting-guidelines/spirit-2013-statement-defining-standard-protocol-items-for-clinical-trials/).

**Table:** 

Title {1}	Parasternal After Cardiac Surgery (PACS): a prospective, randomised, double-blinded, placebo-controlled trial study protocol for evaluating a continuous bilateral parasternal block with lidocaine after open cardiac surgery through sternotomy
Trial registration {2a and 2b}.	The study was registered in the European Clinical Trials Database on 27/9/2019 (registration number: 2018-004672-35).
Protocol version {3}	Amended version 3.1, November 2019
Funding {4}	ML received financial support for this study from Grünenthal Sweden AB (grant number: 2-2191/2020). ML received a personal research award from the Swedish Association for Cardiothoracic Anesthesiology and Intensive Care, donated by CSL-Behring. US is supported by the Swedish Heart−Lung Foundation (grant number: 20190533). AFC is supported by an independent donation from Mr Fredrik Lundberg.
Author details {5a}	Mark Larsson (ML)^1,3^, Ulrik Sartipy (US)^1,4^, Anders Franco-Cereceda (AFC)^1,4^, Anders Öwall (AÖ)^1,3^, Jan Jakobsson (JJ)^2,5^ ^1^ Department of Molecular Medicine and Surgery, Karolinska Institutet, Stockholm, Sweden ^2^ Institution for Clinical Sciences, Karolinska Institutet at Danderyds Hospital, Stockholm, Sweden ^3^ Function Perioperative Medicine and Intensive Care, Section for Cardiothoracic Anaesthesia and Intensive Care, Karolinska University Hospital ^4^ Department of Cardiothoracic Surgery, Karolinska University Hospital, Stockholm, Sweden ^5^ Department of Anaesthesia and Intensive Care, Danderyds Hospital, Stockholm, Sweden **Roles and responsibilities:** Academic sponsor: JJ Principal investigator: ML Study design: ML, AÖ and JJ Collection and management of data: ML Data analysis and interpretation: ML, US, AFC, AÖ and JJ Writing of the report: ML, US, AFC, AÖ and JJ
Name and contact information for the trial sponsor {5b}	Jan Jakobsson, Karolinska Institutet, Sweden. E-mail: jan.jakobsson@ki.se
Role of sponsor {5c}	JJ is the academic sponsor of this study, and is fully involved in planning and designing the study, and analysing the results and writing the report. The funding sources had no role in the design of this study and will not have any role in its execution, analyses, interpretation of the data, preparation of the manuscript or the decision to submit results.

## Introduction

### Background and rationale {6a}

After open heart surgery, patients experience moderate to severe pain [1–4]. Apart from causing acute suffering, severe postoperative pain increases the risk for developing chronic postsurgical pain, which results in a decreased health-related quality of life [2, 5–10]. Pain and its main treatment in cardiac surgery, opioids, can impede rapid recovery. Pain hampers mobilisation and physiotherapy. Opioids affect recovery through negative side effects, such as sedation, nausea, dizziness, respiratory depression and gastrointestinal slowing.

Enhanced recovery (fast track) pathways seek to attenuate the negative effect that surgery has on normal physiology, with the ultimate goal to allow a more rapid return to normal life [11, 12]. A cornerstone of these pathways is effective pain treatment with an opioid-sparing strategy, usually designated multimodal analgesia [13–15]. Nerve blocks using local anaesthetics that block the noxious nerve impulses from reaching the central nervous system can be an effective part of such a strategy. Depending on what level the afferent nerve system is blocked, central and peripheral blocks can be used. In cardiac surgery, a continuous epidural infusion is a central block that offers effective pain treatment [16–19]. However, the fear of epidural haematoma, potentially causing para- or tetraplegia, has limited the use of epidural infusion. Nerve blocks that can be performed at increasingly peripheral levels, such as intercostal, (para)sternal and fascial plane blocks, and even wound infiltration, have been described [20–24]. These nerve blocks can be performed as a single shot and as a continuous block. Because cardiac surgery causes considerable pain that lasts several days, continuous infusion of a local anaesthetic, offering analgesia over an extended period, is advantageous.

Two continuous peripheral blocks that have been described after sternotomy and are anatomically in proximity are sternal wound infiltration and a bilateral sternal block. In sternal wound infiltration, a catheter for continuous local anaesthetic infusion is placed centrally in the sternal wound. A bilateral sternal block is performed by bilaterally inserting a catheter over the costosternal margin. As described by Eljezi et al [25], the aim for a local anaesthetic effect is the anterior branches of the intercostal nerves [26]. Studies that have described these two blocks used either bupivacaine or ropivacaine, both of which are long-acting local anaesthetics [21, 22, 24–32]. The reported results of these studies vary, where several studies did not show any additional analgesic effect beyond standard care, while others showed lower pain scores and lower opioid use. This range of efficacy could be due to the differences in anatomical positioning of the catheter(s). Another possible explanation for the varying study results was provided by White et al [32]. When they compared sternal wound infusion of 0.25% and 0.5% solutions of bupivacaine with each other and to saline, as intuitively expected, they found a greater analgesic effect of the solution with the higher concentration of bupivacaine. However, this finding is contradicted by the results of several other studies. These other studies described the continuous administration of bupivacaine or ropivacaine with doses ranging from 3.75 to 25 mg/hour and from 8 to 13.5 mg/hour, respectively. Several studies [22, 24, 25, 28, 32] that used doses at the lower end of the range described significant effects, while some studies [27, 31, 33] that used higher doses did not.

An increased risk of infection has been raised as a possible issue for continuous regional anaesthesia in cardiac surgery. In a prematurely stopped trial reported by Agarwal et al [27], a continuous infusion of ropivacaine through two multiport catheters placed in the sternal wound was compared with saline. They found a possible higher risk for sternal infection in the active treatment group. Therefore, the choice of the local anaesthetic might play a role, where bupivacaine and lidocaine show more antimicrobial activity than ropivacaine [34].

We consider that the above-mentioned studies show the potential of a continuous peripheral parasternal block after cardiac surgery, but its full effect has not been shown. In an attempt to further investigate this continuous block, we propose examining the use of a short-acting local anaesthetic of lidocaine, which will allow a dose increase for effect and simultaneously address the concern of an increased risk of infection.

### Objectives {7}

We plan to evaluate the analgesic efficacy of the least toxic local anaesthetic lidocaine in a continuous bilateral parasternal block after sternotomy for cardiac surgery. By using a relatively low 0.5% concentration at a total rate of 14 ml/hour (7 ml/hour on each side), our aim is to achieve a spread of local anaesthetic that will effectively block the distal endings of intercostal nerves. We will study whether this block offers effective analgesia and minimises the requirement for opioids. Additionally, we will evaluate if effects can be seen in quality of recovery in a broader perspective after this type of major surgery.

### Trial design {8}

This study will be conducted as a single-centre, randomised, double-blinded, clinical trial.

## Methods: Participants, interventions and outcomes

### Study setting {9}

The study population will consist of adult patients undergoing elective open cardiac surgery at the Karolinska University Hospital in Stockholm.

### Eligibility criteria {10}

The inclusion criteria are as follows: (1) male and non-pregnant female patients aged 20–80 years; (2) American Society of Anesthesiologists Physical Status Classification (ASA) II–II; (3) not undergoing emergency or planned redo surgery; not suffering from (4) severe left heart failure, (5) respiratory insufficiency, (6) advanced kidney failure or (7) pronounced hepatic disease; (8) no allergy to local anaesthetics; (9) no psychiatric disease or any psychoactive medication; (10) no cognitive disturbance and/or inability to understand written and/or oral instructions; (11) no history of chronic pain or chronic pain medication; and (12) informed consent.

The exclusion criteria are as follows: (1) failure to be extubated within 4 hours after surgery; and (2) deep hypothermia during surgery.

If study participants are subject to acute redo after the primary procedure and can be extubated within 4 hours thereafter, the study will continue per protocol.

### Who will take informed consent? {26a}

The principal investigator (ML) will obtain written informed consent from each study participant after oral and written information about the study has been provided to the participants.

### Additional consent provisions for collection and use of participant data and biological specimens {26b}

The study participant information form contains specific information on the collection, handling and analyses of blood samples taken during the trial. The consent form has specific boxes to tick for approval of the collection, handling and analyses of blood samples as explained in the information for the study participants.

## Interventions

### Explanation for the choice of comparators {6b}

This trial is designed to compare a continuous parasternal block with the standard of care. Therefore, the comparator is saline. The standard treatment consists of paracetamol and a patient-controlled analgesia (PCA) pump with morphine.

#### Intervention description {11a}

##### Placement of parasternal catheters and the start of the study intervention

At the end of surgery, after sternal closure and wound closure, the skin surrounding the sternal wound will once again be swabbed with chlorhexidine. Thereafter, the surgeon who performed the procedure will insert a 19-cm multiply perforated catheter subpectorally on either side of the sternum over the costosternal margin. Patients will be randomised to receive either saline 9 mg/ml (0.9%) or lidocaine 5 mg/ml (0.5%). After a bolus from two pre-prepared 20-ml syringes with the allocated solution in each catheter, both catheters will be connected to the elastomeric pump for continuous infusion of the randomised treatment at a rate of 7 ml/hour in each catheter. The resulting lidocaine administration will be 70 mg/hour (1680 mg in 24 hours). The elastomeric single-use pump will be replaced by a new after 24 and 48 hours to attain 3 days of treatment.

##### Analgesic regimen

Preoperatively, patients will receive instructions on the assessment of perceived pain. The instructions are to score perceived pain from 0 to 10 according to the numerical rating scale (NRS), where an NRS score of 0 indicates no pain, and an NRS of 10 is the worst imaginable pain. Pain is further explained and categorised as mild (NRS scores of 1–3), moderate (NRS scores of 4–6) or severe (NRS scores of 7–10).

##### Intensive care unit (ICU)/recovery

Starting at the end of surgery, all patients will receive intravenous or oral paracetamol 1000 mg every 6 hours. After extubation, when the patient states a score greater than mild (an NRS score > 3), the attending ICU/recovery nurse will provide rescue analgesic as follows: (1) an intravenous bolus of morphine will be administered with 1-mg increments up to a total dose of 0.15 mg/kg; (2) if NSAID is tolerated and considered acceptable regarding chest tube flow, 15–30 mg of ketorolac will be administered intravenously; and (3) if tolerated and considered acceptable regarding alertness and blood pressure, 15–75 μg of clonidine will be administered intravenously. All rescue medication can be repeated as considered necessary to achieve an NRS score ≤ 3.

After mild or no pain has been achieved (NRS score ≤ 3), a PCA pump will be connected to allow the patient to perform self-administration of intravenous morphine. The instructions for use will be to achieve an NRS score ≤ 3. On the patient’s demand, the PCA pump will deliver 1 mg of morphine, with a lockout time of 6 minutes and a maximal dose of 30 mg every 4 hours. Rescue analgesia consists of the same algorithm as the one described for rescue medication before starting the PCA pump.

##### Surgical ward

Analgesia after discharge from the ICU will consist of morphine by PCA and oral paracetamol 1000 mg every 6 hours. To treat break-through pain at the surgical ward that cannot be relieved with this regimen, additional intravenous morphine can be administered by the attending nurse. If required, rescue pain treatment will consist of an oral nonsteroidal anti-inflammatory drug (naproxen 250–500 mg) and/or oral clonidine (75 μg).

##### End of parasternal infusion

After the 72-hour study period, the bilateral parasternal catheters will be removed and the PCA pump will be discontinued. If continued analgesia is required after the intervention, the patient will receive the following oral analgesia. (1) Slow-release oxycodone will be administered twice daily. An oral daily oxycodone dose will be approximated to the equivalent of the intravenous dose of morphine administered by the PCA pump during the last 24 hours of the intervention. (2) Paracetamol 1 g will be provided four times daily and rescue medication that consists of short-acting oral oxycodone will be provided.

#### Criteria for discontinuing or modifying allocated interventions {11b}

##### Premature discontinuation of parasternal infusion

Parasternal infusion of the trial medication will be discontinued earlier than after 72 hours for the following reasons: (1) the trial subject retracts consent; (2) inadvertent removal of one or both parasternal catheter(s); (3) surgical complications requiring the removal of one or both parasternal catheter(s); (4) unforeseen medical occurrence caused by one or both parasternal catheter(s); and (5) unforeseen severe and potentially life-threatening medical event that is suspected to be the result of an allergy to or systemic toxicity of the infused study medication.

##### Adverse events that will allow continuation of the infused study medication

Mild to moderate symptoms of lidocaine toxicity such as tinnitus, dizziness, blurred vision, metallic taste, peri-oral numbness and tongue paraesthesia may be managed with medication and are not necessarily reason for discontinuation of the intervention.

##### Premature discontinuation of follow-up

Study participants who cannot engage in the follow-up owing to a prolonged ICU care will be considered dropouts. Further assessment will be discontinued.

### Strategies to improve adherence to interventions {11c}

The intervention will last the first 72 hours after surgery. Study participants will be under continuous care at the ICU/surgical ward and will be visited daily by the study team.

### Relevant concomitant care permitted or prohibited during the trial {11d}

All study participants will receive standard postoperative care according to departmental practice.

### Provisions for post-trial care {30}

At the end of the intervention, the study participants will be cared for according to departmental practice. If required, analgesia will be provided as described above (“Intervention description” subsection). Open cardiac surgery and participating in this trial carry risks. In case any adverse event occurs, our department and the hospital will provide care, irrespective of the cause. The National Patient Safety Insurance will cover potential insurance claims.

### Outcomes {12}

#### Primary endpoint and secondary endpoints

The primary endpoint is cumulated intravenous morphine administration by PCA at 72 hours. The secondary end points are as follows: (1) the cumulated NRS score three times daily at 72 hours; (2) the cumulated NRS score after two deep breaths three times daily at 72 hours; (3) the NRS score at rest and after two deep breaths at 2, 4, 8 and 12 weeks after surgery; (4) oxycodone requirement at 2, 4, 8 and 12 weeks after surgery; (5) Quality of Recovery (QoR)-15 score preoperatively and at 24, 48 and 72 hours, as well as at 2, 4, 8 and 12 weeks after surgery; (6) peak expiratory flow (PEF) preoperatively, postoperatively on the morning after surgery and in the evening from day after surgery until postoperative day 3; and (7) serum concentrations of interleukin-6 (IL-6) and lidocaine at 1, 24, 48 and 72 hours after starting the intervention compared with preoperative values.

#### Other endpoints

In addition to the primary and secondary endpoints, we will observe (1) the incidence of nausea and/or vomiting at any time during the initial 72 hours; (2) the incidence of sedation at any time during the initial 72 hours; (3) the incidence of arrhythmia, with more than single or solitary coupled supraventricular and ventricular extra beats at any time during the initial 72 hours; (4) NRS scores at rest at all time points during the initial 72 hours; (5) morphine consumption at 24, 48 and 72 hours; (6) NRS scores after two deep breaths three times daily during 72 hours; (7) the day of discharge and discharge time; and (8) the occurrence of sternal wound infection.

##### Participant timeline {13}

See Appendix 2.

##### Sample size {14}

Historical data at our institution show a mean (± standard deviation) intravenous morphine requirement of 67 (± 30 mg) after open cardiac surgery for the first 48 postoperative hours. We have previous experience of a continuous bilateral parasternal block after cardiac surgery. When infusing 8 mg ropivacaine/hour in each catheter (2 mg/ml, 4 ml/hour in each catheter), we found a mean morphine requirement of 53 mg the first 2 postoperative days, which equals to a decrease of 14 mg morphine. We believe that the use of a higher equivalent dose of lidocaine will increase efficacy, which we hypothesise will double the decrease in morphine requirement. If we assume a requirement for ≤ 40 mg morphine for the first 48 postoperative hours for patients receiving a bilateral parasternal block, 21 patients in each arm are required to identify a significant difference (*p* < 0.05) with a power of 80%. This sample size calculation is based on the first 48-hour opioid requirement postoperatively. The study participants will be cared for in-hospital for at least 4 days postoperatively. Therefore, we expect a low drop-out for our primary endpoint of opioid requirement at 72 hours. To compensate for potential drop-out and/or incomplete collection of data, 45 patients will be included and randomised.

##### Recruitment {15}

Study participants will be recruited from patients who undergo non-redo open cardiac surgery at our department. We believe that we will be able to recruit an average of two study participants each week. Taking into account holidays and potential logistic problems in the supply of study medication, we aim to recruit all 45 patients within 1 year from starting enrolment.

### Assignment of interventions: allocation

#### Sequence generation {16a}

Because of practical reasons regarding the supply of trial medication, study participants will be randomised in a 1:1 fashion in blocks of eight, except for the last block of five. Each participant will receive an inclusion number, and each inclusion number will correspond to a number on a sealed opaque envelope. Each envelope will contain a random assignment to one of the two treatment groups. A study participant’s name and assigned treatment will be recorded in a randomisation list.

#### Concealment mechanism {16b}

One of two anaesthesia nurses not involved in the care of the study participants and beyond randomisation not involved in this study will open a sealed opaque envelope for allocating treatment.

#### Implementation {16c}

The allocation of treatment (saline vs lidocaine) will be determined by the randomisation as described above. The principal investigator (ML) will prepare the envelopes for allocation. Concealment of allocation will be achieved because an anaesthesia nurse who is not involved in the study will open the envelopes and prepare the study medication. ML will enrol all study participants.

### Assignment of interventions: Blinding

#### Who will be blinded {17a}

The study participants, investigators, participating research nurses, attending anaesthesiologist, anaesthesia nurse and ICU/recovery nurses and nurses on the ward will be blinded to the allocation.

#### Procedure for unblinding if needed {17b}

In case a serious adverse event occurs that requires knowledge of the allocated treatment, the label concealing the content of the elastomeric pump can be removed with some effort. The randomisation list is kept locked in our local thoracic anaesthesia pharmacy and can be made available in case of emergency at any time of the day by contacting the principal investigator.

### Data collection and management

#### Plans for assessment and collection of outcomes {18a}

##### Analgesia

The analgesics administered during surgery and the initial time at the ICU/recovery will be recorded and added to the case record form (CRF). After starting the PCA pump according to the protocol, administered doses will be registered in the PCA pump. The number of provided doses for every 24 hours and the total 72-hour period will be retrieved and recorded in the CRF. If any other rescue medication is required for pain control, the type and dose will be retrieved from the charts.

##### Mobile platform

Push notices with a link to an online platform will be sent to the study participant’s mobile phone at pre-determined times. After two-step verification, access can be gained to the current questionnaire. Only the principal investigator and study nurses will - after two-way verification - have access to the completed questionnaires. For each questionnaire at a certain follow-up time, a separate push notice with a link will be sent. Questionnaires will be sent at specific times to determine the following: (1) QoR-15 score preoperatively and at 24, 48, 72 hours, as well as 2, 4, 8 and 12 weeks after surgery; (2) pain according to the NRS at rest and after two deep breaths, every 8 hours for 72 hours starting the evening after surgery; (3) pain in the evening according to the NRS at 2, 4, 8 and 12 weeks after surgery; (4) requirement of oral oxycodone in the morning and evening at 2, 4, 8 and 12 weeks after surgery; (5) whether any nausea is experienced, every 8 hours for 72 hours starting in the evening after surgery; and (6) whether the trial subject has experienced nausea and/or vomited in the last 8 hours (yes/no), every 8 hours for 72 hours starting in the evening after surgery.

##### Blood samples

Blood samples will be drawn before starting surgery and at 1, 24, 48 and 72 hours after the administration of the study medication bolus dose and the initiation of parasternal infusion. At the specified times, two samples will be drawn. One sample will be sent for direct analysis of IL-6 concentration at the hospital laboratory. The other sample will be centrifuged at 2000 g for 10 minutes. The serum will be pipetted in micro tubes and stored at −70°C. After conclusion of the enrolment, lidocaine concentrations in the serum samples will be determined.

##### PEF

PEF will be measured with a standard peak flow meter. To improve accuracy, we will record the results of three consecutive measurements. PEF will be recorded preoperatively and at each postoperative day until postoperative day 3.

##### Remaining data

Information on weight, length, the discharge time and the length of stay will be obtained from the electronic patient dossier.

#### Plans to promote participant retention and complete follow-up {18b}

The intervention will occur during the first 72 hours after surgery and will be monitored closely by the study team. We hope that our digital platform will facilitate completion and result in a high response rate of the follow-up questionnaires during the 3 months after surgery.

#### Data management {19}

##### Source document

All study data will be retrieved and documented in CRFs, which are only identifiable by a randomisation number.

### Confidentiality {27}

The randomisation key and CRFs will be stored separately. Data labelled with the randomisation number only will be gathered in a file for data management and analyses. These files will be stored at secure servers at the Karolinska Institute or in personal lap-tops provided by the Karolinska Institute and accessible only with personal passwords.

### Plans for collection, laboratory evaluation and storage of biological specimens for genetic or molecular analysis in this trial/future use {33}

Blood samples will be drawn as described above. The collected serum will be stored at −70°C, and will only be identifiable by a randomisation number. After conclusion of the enrolment, lidocaine concentrations will be measured in serum samples. No samples will be saved after this analysis.

### Statistical methods

#### Statistical methods for primary and secondary outcomes {20a}

Effect variables will be analysed per protocol for efficacy. Per protocol is defined as fulfilment of the 72-hour parasternal infusion. The intention-to-treat population will also be analysed, mostly for safety and side effects, but also for efficacy. The safety population will include any patient who received parasternal catheters.

The primary effect variable (cumulated intravenous morphine administered by a PCA pump at 72 hours) will be analysed as a continuous variable. Assessment for normal distribution will be performed by the Kolmogorov–Smirnoff test, Shapiro–Wilk test and Q-Q plot.

Non-parametric statistics will be applied for the secondary effect variables comprising NRS scores, the oxycodone dose and QoR-15 scores. PEF and stress marker levels will be analysed as continuous variables.

Data will be reported as the mean and standard deviation unless otherwise stated. The comparison of normally distributed variables will be performed by Student’s t-test and/or analysis of variance. Variables that do not follow a normal distribution will be analysed by the Wilcoxon test. Categorical data will be analysed with the chi-square test or contingency tables as appropriate. Appropriate statistical tests will be performed for repeated measures. Data management and statistical analysis will be performed using Stata (StataCorp LP, College Station, TX, USA) and R (R Foundation for Statistical Computing, Vienna, Austria).

### Interim analyses {21b}

No interim analyses are planned.

### Methods for additional analyses (e.g. subgroup analyses) {20b}

No additional analyses are planned.

### Methods in analysis to handle protocol non-adherence and any statistical methods to handle missing data {20c}

Protocol non-adherence for opioid requirement will be handled by transforming any opioid other than morphine to a morphine-equivalent value. If there are missing NRS and QoR-15 scores, we will apply last observation carried forward analysis.

### Plans to give access to the full protocol, participant level-data and statistical code {31c}

This is an investigator-initiated trial. There are no publication restrictions. The funders have no role in the study design, data collection and analysis, preparation of the manuscript or decision to publish.

### Oversight and monitoring

#### Composition of the coordinating centre and trial steering committee {5d}

This is a single-centre trial. No steering committee will be formed. The principal investigator (ML) will inform the sponsor (JJ) on a weekly basis on the progress of the trial.

#### Composition of the data monitoring committee, its role and reporting structure {21a}

No data monitoring committee will be formed because the trial will only encompass 45 patients and is planned to be completed within 18 months from starting the enrolment.

#### Adverse event reporting and harms {22}

During the intervention, the principal investigator (ML) will actively seek and be informed on a daily basis of any adverse event occurring. During the follow-up time after discharge until 12 weeks after surgery, the QoR-15 questionnaire will provide information on adverse events. We will monitor for possible re-intake of study participants regarding adverse events. The sponsor (JJ) will be informed about all and any adverse events. Serious adverse events will be reported within 24 hours.

All adverse events will be recorded in the CRF. A distinction will be made between expected and unexpected adverse events. Each adverse event will be assessed for the intensity (mild, intermediate or severe), severity (serious vs non-serious) and relationship with the administered trial medication (non-related, possibly related or probably related). Serious adverse events will be presented to and discussed with all investigators within 1 week of being identified.

All adverse effects will be reported in accordance with the applicable local and European Union laws and regulations. The Swedish Medical Products Agency, Swedish Ethical Review authority and all participating investigators will be informed in accordance with the timelines set out in the applicable laws and regulations.

### Frequency and plans for auditing trial conduct {23}

We plan for an independent research nurse who is not involved in the trial and not working at the same site as the investigators to perform an audit during the trial. The audit will review good clinical practice adherence, enrolment, consent and data reporting in the CRF.

### Plans for communicating important protocol amendments to relevant parties (e.g. trial participants, ethical committees) {25}

No changes to the protocol and procedures stated will be undertaken unless the Swedish Ethical Review authority, and if applicable, the Swedish Medical Products Agency are informed and have approved the amendment.

### Dissemination plans {31a}

The results of the trial will be presented in a peer-reviewed publication. No later than 3 years after the completion of the last study participant’s 12-week follow-up, deidentified participant-level data will be made available in an appropriate data archive for the purpose of sharing.

## Discussion

The objective of this trial is to determine if a continuous bilateral parasternal block with continuous infusion of lidocaine reduces intravenous morphine consumption after open heart surgery. This issue is clinically important because a decreased need for postoperative opioids is thought to enhance patients’ recovery following cardiac surgery.

To enable a parasternal block to reach its full analgesic potential, we believe that the large area (i.e., the number of nerves) that needs to be blocked requires a larger amount (dose) of local anaesthetic. Eljezi and D’Ostrevy showed a visually adequate spread of local anaesthetic when they injected a bolus of 20 ml in a parasternal catheter [26]. A local anaesthetic should also reach a sufficiently high concentration at the site of effect to fully block nerve conduction. We believe that the increased effect of a higher concentration of bupivacaine in a study by White et al [32] reflects this view. However, the highest recommended doses of bupivacaine and ropivacaine limit the use of a larger dose to increase the effect. The maximally recommended daily dose for bupivacaine is 400 mg (equivalent to 16.7 mg/hour) and that for ropivacaine is 675–800 mg (equivalent to 28–33.3 mg/hour). The risk of a toxic reaction to a local anaesthetic increases with the dose and subsequent higher plasma concentrations. As noted by White et al [32], while similar serum bupivacaine concentrations were found at 24 hours, the patients who were randomised to the 0.5% solution had higher serum bupivacaine concentrations at 48 hours than patients who received the 0.25% solution. Both solutions were infused at a rate of 4 ml/hour. Nasr et al [22], who infused 0.25% bupivacaine at a rate of 5 ml/hour for 48 hours, also showed an increase in plasma bupivacaine concentrations from 24 to 48 hours. Neither White et al [32] nor Nasr et al [22] found toxic plasma concentrations. In contrast, in Eljezi et al’s [29] study on the use of bilateral sternal block in high-risk cardiac surgery patients, they showed an increase in ropivacaine plasma concentrations over time that reached potentially toxic plasma concentrations in 22.2% of patients. Notably, despite these high plasma concentrations, none of the trial subjects experienced toxic symptoms.

These findings suggest that uptake and potential systemic toxicity are reasons for caution when considering high doses of long-acting local anaesthetics to achieve the optimal analgesic effect. When infused over a longer period of time, accumulating long-acting local anaesthetics have an increased risk of toxic plasma concentrations.

To address the risk of toxic plasma/serum concentrations when long-acting local anaesthetics are infused, especially when the dose is increased to improve efficacy, we suggest the use of lidocaine as an alternative. Lidocaine is a well-studied, frequently used, short-acting local anaesthetic, and it also has an established intravenous indication as an anti-arrhythmic. Additionally, intravenous infusion of lidocaine has been extensively studied. Several reviews have evaluated its postoperative analgesic effect, one has assessed the treatment of other acute pain [35–39]. In these studies, intravenous lidocaine was administered as a bolus followed by a continuous infusion at doses of 1 to 5 mg/kg/hour. The duration of infusion varied greatly. With regard to pain treatment after cardiac surgery, one study has evaluated intravenous lidocaine [40]. In this study, an initial bolus of 1.5 mg/kg was followed by a 48-hour continuous lidocaine infusion of 30 μg/kg/minute (1.8 mg/kg/hour), which resulted in a dose of 3024 mg/24 hours for a 70-kg person. This intravenous infusion resulted in serum lidocaine concentrations between 2 and 5 μg/ml, but they did not decrease the postoperative fentanyl requirement or improve pain scores.

In addition to the analgesic effect of lidocaine, it has an anti-inflammatory effect. Although the exact mechanisms of lidocaine are unknown, they affect multiple steps in the inflammatory response [41]. Clinical studies have shown signs of a decreased inflammatory response in lidocaine treatment. In a study on asthma patients, inhaled lidocaine showed a positive treatment effect which was comparable to glucocorticoids [42].. After hysterectomy, lower systemic pro-inflammatory cytokine concentrations were observed when lidocaine was infused intravenously during surgery [43]. In cardiac surgery, as a possible sign of a reduced reperfusion inflammatory response, intravenous lidocaine decreased myocardial injury markers after coronary bypass surgery [44]. However, intravenous lidocaine has not shown any certain neuroprotective effect in this patient group [45–47].

We are aware that the analgesic regimen that we propose allows for several different analgesics with different routes of administration to be used as rescue medication. Despite the risk that this regimen might complicate our evaluation of the parasternal block, we believe that this multimodal approach is our clinical standard and should be offered as a possible treatment to our study participants.

In conclusion, we hypothesise that a continuous bilateral parasternal block with infusion of lidocaine 5 mg/ml at a rate of 7 ml/hour on each side (resulting in a total dose of 70 mg lidocaine/hour) will result in lower opioid requirement postoperatively and lower pain scores during the first 72 hours following open heart surgery. We aim to evaluate whether a potentially decreased opioid requirement and pain in addition to a possible anti-inflammatory effect of lidocaine in plasma will result in an enhanced quality of recovery. We recognise that a continuous infusion of lidocaine will result in its presence in plasma and have taken into consideration recommendations for intravenous dosing in our study design.

### Trial status

The recruitment of this trial was initiated in June 2021, and we are currently enrolling patients. The protocol that we adhere to is the amended version v 3.1, June 2019. We expect to complete enrolment in April 2022. The follow-up will be finalised in July 2022.

## Supplementary Information

**Additional file 1.**

**Additional file 2.**

## Data Availability

The sponsor and all investigators will have full access to the final trial dataset.

## Abbreviations

CRF: case record form
IL-6: interleukin-6
NRS: numerical rating scale
PACS: Parasternal After Cardiac Surgery
PCA: patient-controlled analgesia
PEF: peak expiratory flow
QoR-15: Quality of Recovery-15
