# Supplementary figures and images for: Parasternal After Cardiac Surgery (PACS): a prospective, randomised, double-blinded, placebo-controlled trial study protocol for evaluating a continuous bilateral parasternal block with lidocaine after open cardiac surgery through sternotomy
Source: Trials. 2022 Jun 20;23:516. doi: 10.1186/s13063-022-06469-5 (PMC9208208; doi:10.1186/s13063-022-06469-5)

# Appendix 1

## Consort Flow Diagram for PACS – Parasternal After Cardiac Surgery

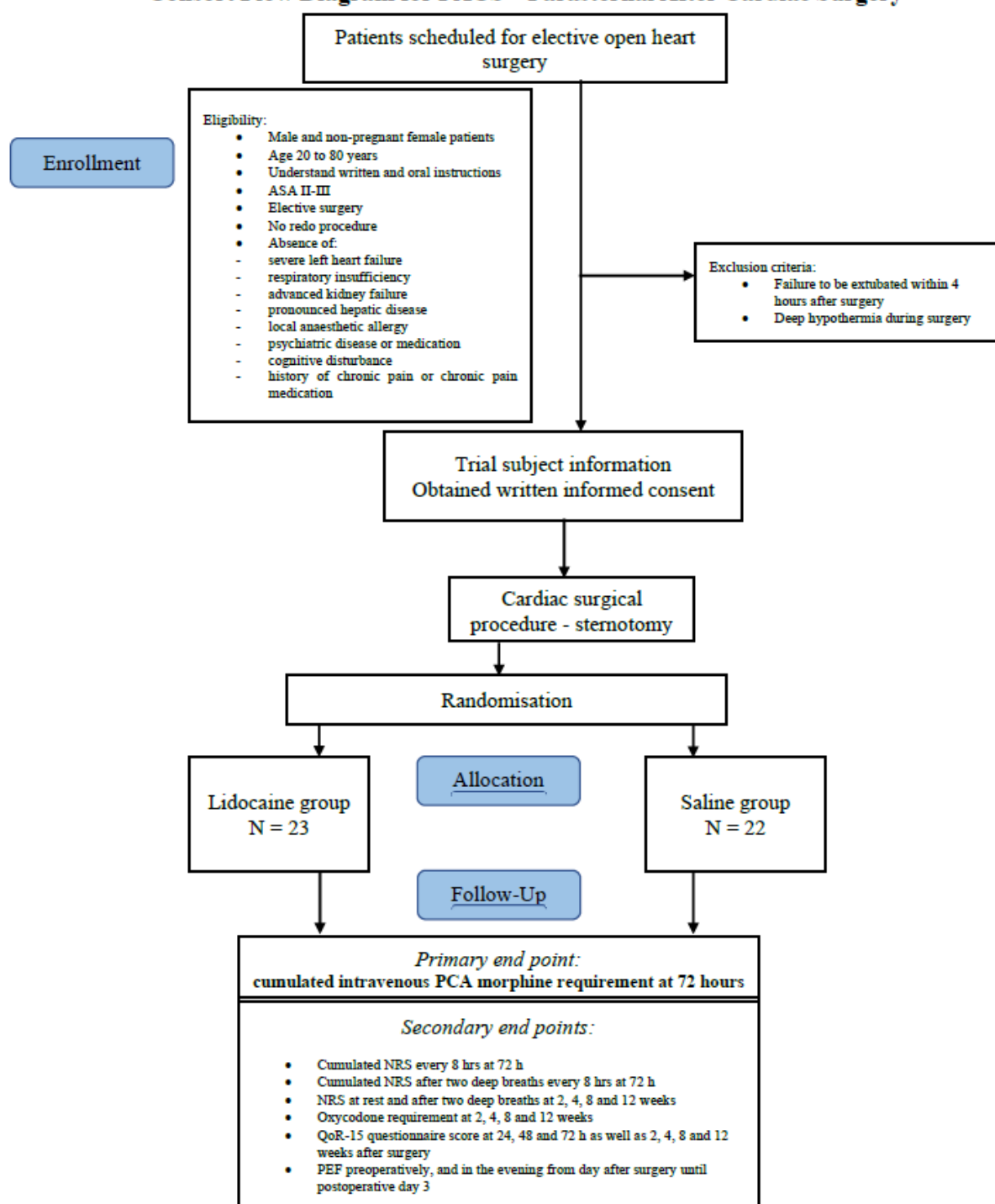

Supplement: Supplementary file 1 — Additional file 1. [file 13063_2022_6469_MOESM1_ESM.pdf]
