# Supplementary material for: Parasternal After Cardiac Surgery (PACS): a prospective, randomised, double-blinded, placebo-controlled trial study protocol for evaluating a continuous bilateral parasternal block with lidocaine after open cardiac surgery through sternotomy
Source: Trials. 2022 Jun 20;23:516. doi: 10.1186/s13063-022-06469-5 (PMC9208208; doi:10.1186/s13063-022-06469-5)
Supplement: Supplementary file 2 — Additional file 2. [file 13063_2022_6469_MOESM2_ESM.pdf]

Appendix 2

PACS trial assessments

Protocol (day)	-8	0 surgery	1	2	3	14	28	56	84
	screening & pre-trial test	parasternal infusion	parasternal infusion	parasternal infusion	follow up	follow up	follow up	follow up	End of study
QoR-15	+		+	+	+	+	+	+	+
PEF	+	+	+	+	+				
Blood test		x 2	x 1	x 1	x 1				
NRS-r?		x 1	x 3	x 3	x 3				
NRS-x?		x 1	x 3	x 3	x 3				
nausea?		x 1	x 3	x 3	x 3				
vomiting?		x 1	x 3	x 3	x 3				
analgesia?		PCA	PCA	PCA	PCA	dose?	dose?	dose?	dose?

QoR-15 = Quality of Recovery – 15 questionnaire; PEF = Peak Expiratory Flow; NRS-r = pain assessment according to Numerical Rating Scale at rest; NRS-x = pain assessment according to Numerical Rating Scale after deep breath/movement; PCA = Patient Controlled Analgesia Pump registration of dose; dose? = oxycodone dose; + = assessment made and registered; x1, x 2, x 3 = number of times assessment made for that day
